# Exploring Pathways between Internalized Weight Bias, Eating Disorder Psychopathology, and Weight Loss Expectations in Treatment-Seeking Adults with Binge Eating and Obesity

**DOI:** 10.21203/rs.3.rs-5357165/v1

**Published:** 2024-11-22

**Authors:** Katrina Obleada, Adrian Ortega, Isabel Rooper, Leah Parsons, Macarena Kruger, Chidiebere Azubuike, Lindsay Lipman, Diane Chen, Jennifer Wildes, Andrea Graham

**Affiliations:** Potocsnak Family Division of Adolescent and Young Adult Medicine, Ann & Robert H. Lurie Children’s Hospital of Chicago; Center for Behavioral Intervention Technologies, Northwestern University Feinberg School of Medicine; Center for Behavioral Intervention Technologies, Northwestern University Feinberg School of Medicine; Center for Behavioral Intervention Technologies, Northwestern University Feinberg School of Medicine; Center for Behavioral Intervention Technologies, Northwestern University Feinberg School of Medicine; Center for Behavioral Intervention Technologies, Northwestern University Feinberg School of Medicine; Center for Behavioral Intervention Technologies, Northwestern University Feinberg School of Medicine; Potocsnak Family Division of Adolescent and Young Adult Medicine, Ann & Robert H. Lurie Children’s Hospital of Chicago; Department of Psychiatry & Behavioral Neuroscience, University of Chicago; Center for Behavioral Intervention Technologies, Northwestern University Feinberg School of Medicine

**Keywords:** binge eating, obesity, weight bias, weight loss

## Abstract

**Purpose:**

Individuals with obesity and binge eating face weight stigma, which can lead to internalized weight bias (IWB), reinforce eating disorder (ED) pathology, and promote unrealistic weight loss expectations (WLE). Greater understanding of pathways between IWB, ED pathology, and WLE could inform interventions to promote healthy WLE and reduce IWB. This study explored pathways through which IWB directly and indirectly relates to eating pathology and WLE in treatment-seeking adults with obesity and recurrent binge eating.

**Methods:**

Participants (*N* = 199, *Mage* = 40.3, *SD* = 14.3) completed the Eating Disorder Examination interview (EDE) and questionnaire (EDE-Q), Modified Weight Bias Internalization Scale, and Positive and Negative Affect Scale. WLE were calculated based on participants’ expected weight loss divided by current weight. We hypothesized that greater IWB would be associated with greater eating pathology and higher WLE. Pearson correlations were examined to identify possible pathways, followed by exploring direct and indirect associations for pathways with significant correlations.

**Results:**

IWB was positively correlated with Eating and Shape Concerns, as well as negative affect (*p* < 0.05), but not with WLE. Negative affect was positively correlated with WLE. In the pathway model, IWB was directly, negatively associated with WLE (*b*=−0.02, *p* < 0.05). Negative affect was a significant indirect pathway between IWB and WLE (*b* = 0.01).

**Conclusions:**

Our results align with previous literature showing that IWB reinforces eating pathology. Interventions targeting negative affect might promote more reasonable WLE.

## 1. Introduction

Behavioral weight loss treatments usually aim to help individuals lose between 5–10% of their body weight [1]. However, individuals presenting for behavioral weight loss treatments typically overestimate how much weight they will lose (> 10%) [2]. Between 13–30% of adults presenting for weight loss treatments also experience binge eating [2]. Voss and colleagues found that, among adults with obesity and binge eating, those with higher rates of binge eating as well as those who more severely overvalued their shape and weight tended to overestimate their expected weight loss in treatment [2]. This is significant given that discrepancies between weight loss expectations (WLE) and actual weight loss have been associated with weight loss dissatisfaction, treatment attrition, and lower weight loss maintenance [2]. For individuals with comorbid binge eating, this discrepancy may lead to worse outcomes, such as shame and exacerbation of binge-eating episodes [3], which can interfere with weight loss. Therefore, addressing weight loss expectations early and directly with treatment seekers may increase their treatment satisfaction, engagement, and outcomes for both binge eating and weight. Doing so must consider how WLE are formed.

Individuals with obesity often face weight stigma and weight-based discrimination, which leads some to impute these social stereotypes and exhibit internalized weight bias (IWB) or self-directed negative beliefs and attitudes based on their perceived weight status [1]. IWB is associated with numerous negative psychological (e.g., depression, anxiety, low self-esteem, distress, eating pathology, fat phobia, appearance evaluation, quality of life) and physical consequences [1, 4–5].

IWB might also impact weight loss expectations. For example, those with greater IWB may have a stronger desire to lose more weight, compared to those with less IWB, given their negative attributions about their weight. Jung and colleagues found that participants who experienced weight discrimination, IWB, and body image concerns exhibited the greatest discrepancies between their current and desired weight [1]. However, the pathways through which IWB might influence or shape WLE are unclear, especially in samples with obesity and binge eating. Understanding and targeting the pathways through which IWB impacts WLE might help treatment-seeking individuals with obesity and binge eating formulate more realistic WLE.

This study sought to fill a research gap by exploring pathways through which IWB directly and indirectly relates to WLE in treatment-seeking adults with obesity and recurrent binge eating. We hypothesized that greater IWB would be directly associated with greater weight loss expectations. Given that IWB is commonly associated with eating pathology and negative emotions, we hypothesized that IWB would be indirectly related to WLE via multiple pathways such as through eating pathology and negative affect.

## 2. Material and methods

This study was a secondary analysis of baseline data collected for a clinical trial that tested the preliminary efficacy of FoodSteps, a digital intervention for reducing binge eating and managing weight. This study was approved by the Northwestern University Institutional Review Board (STU00208056).

### 2.1 Participants & Procedures

Participants were recruited via flyers posted in Chicago, IL, ResearchMatch, social media advertisements, and word-of-mouth referrals. A total of 1,557 interested individuals consented to and completed an online screener to assess their initial eligibility. To be eligible for the baseline assessment, participants needed to self-report being an English-speaking, non-pregnant adult (≥ 18 years old) with recurrent binge eating (≥ 12 objectively large binge episodes in the past 3 months) and obesity (Body Mass Index [BMI] ≥ 30 kg/m^2^). Eligible respondents also needed to report distress over their binge eating episodes and an interest in using a digital intervention to reduce their binge eating and weight. Exclusion criteria at screening included an unstable medication dosage (taking medication for binge eating or weight management for less than 1 month) or concurrent enrollment in clinical services for weight management or binge eating (e.g., medically supervised weight loss program, psychotherapy).

Of the 357 potentially eligible screens, 199 participants completed the baseline assessment, which was conducted virtually via Zoom. During the baseline assessment, participants provided verbal consent and completed the Eating Disorder Examination (EDE) clinical interview. After the assessment, participants were sent questionnaires via a Research Electronic Data Capture (REDCap) survey link. Participants received $10 compensation for completing the baseline assessment.

### 2.2. Measures

#### 2.2.1. Demographics. Participants self-reported their age, gender identity, race/ethnicity, and income.

#### 2.2.2. Internalized weight bias.

Participants completed the 11-item Modified Weight Bias Internalization Scale (WBIS-M), which assesses the degree to which respondents have accepted negative weight-related stereotypes as personally relevant and true (α = 0.88 in this sample) [5]. Respondents rated the extent of agreement with each item”) on a 7-point scale (1: “*strongly disagree*”; 7: “*strongly agree*”).

#### 2.2.3. Eating pathology.

A trained assessor administered the EDE interview (Version 17), to assess binge eating and compensatory behaviors [6]. Participants also completed the EDE-Questionnaire 6.0 (EDE-Q) [7], a widely used 28-item self-report adaptation of the EDE interview. The EDE-Q uses a 7-point rating scale (e.g., 0: “*no days*” or “*not at all*”; 6: “*every day*” or “*markedly*”) and generates 4 subscale scores: Restraint (α = 0.82), Eating Concerns (α = 0.76), Shape Concerns (α = 0.78), and Weight Concerns (α = 0.46); however, we did not include the Weight Concerns subscale in our study given its unacceptable internal consistency.

#### 2.2.5. Affect.

Participants completed the Positive and Negative Affect Schedule (PANAS), which asked respondents to rate the extent to which they experienced each of 20 mood states (e.g., “*excited*,” “*nervous*”) over the past week using a 5-point scale (1: “*very slightly or not at all*”; 5: “*extremely*”) [8]. We calculated the Negative Affect subscale (α = 0.89) from this measure.

#### 2.2.6. Anthropometrics.

We calculated BMI for each participant using the imperial equation ([weight (lb)] / [height (in)]^2^ × 703) from participants’ self-reported height on the EDE-Q and a weight from a verified photo participants uploaded via REDCap survey.

#### 2.2.7. Weight loss expectations.

We calculated WLE as a percentage of participants’ expected weight loss (“*What amount of weight loss do you think would be reasonable and/or achievable for you in four months?*”) divided by their current weight [2]. Similar to Voss and colleagues, we imputed WLE values for 12 participants who reported a WLE that was reasonably assumed to be their goal weight rather than their expected weight loss (e.g., an expectation to lose 200 pounds of their current weight of 253 pounds) [2]. In these cases, we substituted the difference between their current weight and reported WLE (e.g., in the example above, the imputed value was 53 pounds) for their WLE.

### 2.3 Analytic Plan

Analyses were conducted using SPSS software (version 28; IBM Corp). We conducted descriptive and frequency statistics for participant demographics and all study measures (Table 1). We first tested correlations between IWB, eating disorder pathology negative affect, and WLE to identify possible pathways between IWB and WLE (Table 2). Variables with significant correlations with both IWB and WLE were selected for pathway testing via regression analyses. Using the PROCESS macro and the Preacher and Hayes (2008) method, we tested direct and indirect pathways between IWB and WLE for each variable selected for pathway testing [9]. We used 1000 bootstrap samples at a 95% confidence interval to calculate direct and indirect associations.

## 3. Results

### 3.1 Descriptive Statistics.

Approximately 71% of participants had a WLE above 10%. There were no significant differences in IWB or WLE between males and females. There was a small correlation between BMI and WLE (*r* = 0.15, *p* = 0.04), but not between BMI and IWB (*r* = 0.08, *p* = 0.30).

### 3.2 Correlational results.

Only negative affect was significantly correlated with IWB and WLE, so this variable was selected for pathway testing (Supplemental Table 3). Notably, however, IWB was significantly correlated with eating and shape concerns as well as negative affect.

### 3.3 Regression results.

IWB was directly and negatively associated with WLE when accounting for negative affect (Table 2). Negative affect also demonstrated significant and direct association with WLE. Negative affect (*b* = 0.01, CI: 0.003–0.02; *β* = 0.16, CI: 0.05–0.26) was a significant indirect pathway between IWB and WLE.

## 4. Discussion

This study examined pathways through which IWB directly and indirectly relates to WLE in treatment-seeking adults with recurrent binge eating and obesity. Our hypothesis that greater IWB would be directly associated with greater WLE was not supported. It is possible that individuals with high IWB may be more self-critical and pessimistic about their abilities to lose weight, thus actually leading to lower WLE. Wang and colleagues found that rumination or perseverative negative thinking was associated with IWB in a sample of adults with obesity and binge eating [10]. It is possible that rumination on negative feelings and weight-related stereotypes about oneself might diminish WLE instead of increasing motivation to lose a lot of weight, as initially hypothesized.

Consistent with the literature, we found that IWB was related to eating pathology such as eating and shape concerns, as well as negative affect [1, 5]. However, only negative affect was directly related to greater WLE, indicating eating pathology concerns alone might not relate to high WLE unless these concerns cause heightened negative emotionality. Indeed, these constructs indirectly linked higher IWB to greater WLE, suggesting that negative affect may explain how IWB relates to WLE.

These findings have clinical implications for treatment-seeking individuals with obesity and binge eating. Internalizing weight stereotypes is associated with disordered eating and negative affect, which potentially makes individuals vulnerable to poor treatment outcomes. Clinicians should screen for IWB and negative affect when working with adults with obesity and binge eating such as in weight management and bariatric surgery clinics. Additionally, treatment should target IWB, such as through acceptance-based interventions [1] and by instilling a focus on health instead of thin ideals. In turn, this might promote more body acceptance and minimize non-health related reasons for weight loss (e.g., outward appearance, desired number on scale) that often lead to unsustainable behavior changes.

### 4.1 Strengths, Limitations, and Future Directions

Strengths of this study include a diverse mixed-gendered, treatment-seeking sample with both binge eating and obesity, which may enhance the generalizability of the findings. However, the study is limited by its cross-sectional design. Future studies should use experimental and longitudinal designs to test how IWB directly and indirectly impacts WLE over time. Additionally, a future study might examine other mechanisms linking IWB and WLE, such as learned helplessness or shame [3].

## 5. Conclusions

Our results align with previous literature showing that IWB is associated with eating pathology and negative affect in a sample of treatment-seeking adults with binge eating and obesity. However, only negative affect was directly related to WLE and was an indirect pathway between IWB and WLE. Interventions targeting IWB might promote a healthier (vs objectifying) view of themselves, reduce negative affect, and increase confidence in their ability to make healthy lifestyle choices. Future studies should incorporate experimental and longitudinal designs to determine directional relationships between these constructs.

## Supplementary Material

Supplement 1

## Figures and Tables

**Table 1:** Participant characteristics

	*N*	Mean (SD) or %
Age	199	42.09 (14.28)
BMI	199	39.16 (8.13)
Total Binge Eating Episodes in past 3 months (Winsorized)	199	52.26 (37.82)
Gender Identity		
Male	48	24.1
Female	148	74.4
Another not listed*	2	1.0
Missing	1	0.5
Race		
Native American or Alaskan Native	4	2.0
Asian	8	4.0
Black or African American	91	45.7
Native Hawaiian or Pacific Islander	1	0.5
White	76	38.2
Multiracial	16	8.0
Missing	3	1.5
Ethnicity		
Hispanic/Latino	50	25.1
Not Hispanic/Latino	147	73.9
Missing	2	1.0
Approximate Annual Household Income	178	73,459 (78,413)
% Weight Loss Expectation	198	15.4 (0.10)
Internalized Weight Bias	179	5.03 (1.28)
EDE-Q Scales		
Restraint	184	1.97 (1.59)
Eating Concern	185	2.90 (1.44)
Shape Concern	185	4.50 (1.08)
Negative Affect	181	30.67 (8.85)

Note. BMI = Body Mass Index; EDE-Q: Eating Disorder Examination-Questionnaire

* This is the exact wording from our demographic survey with the option for participants to specify their gender identity.

**Table 2:** Model coefficients for regression analyses of internalized weight bias and negative affect predicting weight loss expectations

Predictor	Outcome							
	Negative Affect				Weight Loss Expectations			
	Coeff.	*β*	*SE*	*p*	Coeff.	*β*	*SE*	*p*
Internalized Weight Bias	3.45	0.50	2.33	<0.01	−0.02	−0.21	0.01	0.02
Negative Affect					0.004	0.33	0.0009	<0.01
Constant	13.36		0.45	<0.01	0.12		0.03	3.9
	*R*^*2*^ = 0.25, *F*(1,174) =58.8, *p*<0.01				*R*^*2*^ = 0.08, *F*(2,173) =7.61, *p*<0.01			

## Data Availability

The datasets generated during and/or analyzed during the current study are available from the corresponding author on reasonable request.

## References

[1] Jung F, Spahlholz J, Hilbert A, Riedel-Heller SG, & Luck-Sikorski C. (2017). Impact of weight-related discrimination, body dissatisfaction and self-stigma on the desire to weigh less. Obesity Facts, 10, 139–151. 10.1159/000468154 28434008 PMC5644951

[2] Voss C, Liu J, Chang A, Kosmas JA, Biehl A, Flynn RL, Kruzan KP, Wildes JE, & Graham AK (2023). Weight loss expectations of adults with binge eating: Cross-sectional study with a human-centered design approach. JMIR Formative Research, 7, e40506. 10.2196/40506 36853750 PMC10015344

[3] O’Loghlen E, Grant S, & Galligan R. (2021). Shame and binge eating pathology: A systematic review. Clinical Psychology & Psychotherapy, 29, 147–163. 10.1002/cpp.2615 34010473

[4] Latner JD, Durso LE, & Mond JM. (2013). Health and health-related quality of life among treatment-seeking overweight and obese adults: Associations with internalized weight bias. Journal of Eating Disorders, 1, 3. http://www.jeatdisord.com/content/1/1/3 24764526 10.1186/2050-2974-1-3 PMC3776203

[5] Pearl RL, & Puhl RM. (2014). Measuring internalized weight attitudes across body weight categories: Validation of the modified weight bias internalization scale. Body Image, 11. 10.1016/j.bodyim.2013.09.005 24100004

[6] Cooper Z, & Fairburn CG. (1987). The eating disorder examination: A semi-structured interview for the assessment of the specific psychopathology of eating disorders. International Journal of Eating Disorders, 6(1), 1–8. 10.1080/21662630.2013.840119

[7] Fairburn CG, & Beglin S. (2008). Eating disorder examination questionnaire. In: Fairburn CG, editor. Cognitive behavior therapy and eating disorders. New York, NY: Guilford Press. pp. 309–313.

[8] Watson D, Clark LA, & Tellegen A. (1988). Development and validation of brief measures of positive and negative affect: The PANAS scales. Journal of Personality and Social Psychology, 54(6), 1063–1070. 10.1037/0022-3514.54.6.1063 3397865

[9] Preacher KJ, & Hayes AF. Asymptotic and resampling strategies for assessing and comparing indirect effects in multiple mediator models. Behavior Research Methods 40, 879–891 (2008). 10.3758/BRM.40.3.879 18697684

[10] Wang SB, Lydecker JA, & Grilo CM. (2017). Rumination in patients with binge-eating disorder and obesity: Associations with eating-disorder psychopathology and weight bias internalization. European Eating Disorders Review, 25, 98–103. 10.1002/erv.2499 28078784 PMC5318238

