# Supplementary material for: Exploring Pathways between Internalized Weight Bias, Eating Disorder Psychopathology, and Weight Loss Expectations in Treatment-Seeking Adults with Binge Eating and Obesity
Source: Res Sq. 2024 Nov 22:rs.3.rs-5357165. Preprint. [Version 1] doi: 10.21203/rs.3.rs-5357165/v1 (PMC11601859; doi:10.21203/rs.3.rs-5357165/v1)
Supplement: Supplement 1 [file NIHPPRS5357165V1-supplement-1.pdf]

# Supplementary Files

This is a list of supplementary files associated with this preprint. Click to download.

- SupplementalTable3.docx
